# Single cell transcriptomics reveals distinct transcriptional responses to oxycodone and buprenorphine by iPSC-derived brain organoids from patients with opioid use disorder

**DOI:** 10.1038/s41380-022-01837-8

**Published:** 2022-10-27

**Authors:** Ming-Fen Ho, Cheng Zhang, Irene Moon, Xiujuan Zhu, Brandon J. Coombes, Joanna Biernacka, Michelle Skime, Tyler S. Oesterle, Victor M. Karpyak, Kristen Schmidt, Kate Gliske, Quyen Ngo, Cedric Skillon, Marvin D. Seppala, Hu Li, Richard M. Weinshilboum

**Affiliations:** 1 Department of Psychiatry and Psychology, Mayo Clinic, Rochester, MN USA; 2 Department of Molecular Pharmacology and Experimental Therapeutics, Mayo Clinic, Rochester, MN USA; 3 Division of Computational Biology, Quantitative Health Sciences; Mayo Clinic, Rochester, MN USA; 4 Hazelden Betty Ford Foundation, Center City, Minnesota USA

**Keywords:** Cell biology, Molecular biology, Neuroscience

## Abstract

The opioid epidemic represents a national crisis. Oxycodone is one of the most prescribed opioid medications in the United States, whereas buprenorphine is currently the most prescribed medication for opioid use disorder (OUD) pharmacotherapy. Given the extensive use of prescription opioids and the global opioid epidemic, it is essential to understand how opioids modulate brain cell type function at the single-cell level. We performed single nucleus RNA-seq (snRNA-seq) using iPSC-derived forebrain organoids from three male OUD subjects in response to oxycodone, buprenorphine, or vehicle for seven days. We utilized the snRNA-seq data to identify differentially expressed genes following drug treatment using the Seurat integrative analysis pipeline. We utilized iPSC-derived forebrain organoids and single-cell sequencing technology as an unbiased tool to study cell-type-specific and drug-specific transcriptional responses. After quality control filtering, we analyzed 25787 cells and identified sixteen clusters using unsupervised clustering analysis. Our results reveal distinct transcriptional responses to oxycodone and buprenorphine by iPSC-derived brain organoids from patients with OUD. Specifically, buprenorphine displayed a significant influence on transcription regulation in glial cells. However, oxycodone induced type I interferon signaling in many cell types, including neural cells in brain organoids. Finally, we demonstrate that oxycodone, but not buprenorphine activated STAT1 and induced the type I interferon signaling in patients with OUD. These data suggest that elevation of STAT1 expression associated with OUD might play a role in transcriptional regulation in response to oxycodone. In summary, our results provide novel mechanistic insight into drug action at single-cell resolution.

## Introduction

Opioid use disorder (OUD) is a disease that, like many other chronic diseases, typically requires long-term treatment and care to prevent relapse [1]. The opioid epidemic is a national crisis that affects public health and the social and economic welfare of the United States. Approximately 130 Americans die every day from an opioid overdose [2]. The number of overdose deaths involving opioids increased six-fold from 1999 to 2017 [2]. Medication-assisted treatment (MAT) should be the first-line treatment for patients with OUD [3]. Buprenorphine, naltrexone, and methadone are the three medications that have received US Food and Drug Administration (FDA) approval for the treatment of OUD [4]. These medications are effective if taken appropriately [3]. However, treatment retention is challenging for OUD patients, which impairs treatment efficacy. A recent report stated that only ~40% of OUD patients treated with buprenorphine/naloxone continued treatment for at least six months (*n* = 27,273) [5]. In addition, a randomized trial comparing extended-release injectable suspension and oral naltrexone showed that OUD patients treated with injection naltrexone displayed much higher treatment retention rates than were seen for oral naltrexone (60% vs. 30%) during 6 months of treatment [6]. A major goal of addiction research is the development and optimization of effective drugs to treat substance use disorders. It would represent a major achievement for addiction medicine if we were to develop ways to reduce exposure to opioids and prevent OUD.

Current in vitro assays and in vivo models designed to study the pathophysiology of OUD and discover potential therapeutic targets are limited. The breakthrough technology represented by induced pluripotent stem cell (iPSC) reprogramming and generation of central nervous system cells and tissues represents a significant step forward. This technology offers a unique opportunity to recapitulate both normal and pathological processes for virtually any human tissue [7]. Brain organoids derived from iPSCs are 3-D self-assembled structures containing multiple brain cell types such as neurons, astrocytes, and microglia [8, 9]. Single-cell sequencing is a new and powerful technology for studying the molecular underpinnings of cellular heterogeneity and the molecular consequences of cellular variability [10, 11]. Therefore, the application of single-cell sequencing of iPSC-derived brain organoids represents an unbiased research tool that can provide information about the cell-type-specific and drug-specific transcriptional response. The utilization of iPSC-derived 3-D brain organoids from OUD patients and modern next-gen sequencing technologies could provide novel insight into OUD’s biologic mechanisms and offer a unique opportunity to develop novel therapeutic agents for OUD treatment.

Oxycodone is one of the most prescribed opioid medications in the United States, whereas buprenorphine is currently the most prescribed medication for OUD pharmacotherapy [12, 13]. This study was designed to identify gene expression profiles associated with drug treatment with oxycodone or buprenorphine drug treatment in iPSC-derived brain organoids from OUD patients. We chose this approach to reveal gene regulatory networks in the biological pathways altered in response to oxycodone or buprenorphine. We also performed functional genomic studies of genes, transcription factors, and pathways identified during this series of experiments. Our subsequent findings enhance the general understanding of drug mechanism(s) of action and the underlying pathophysiology responsible for opioid addiction, thus opening new avenues for discovering novel therapeutic targets for the treatment of OUD.

## Methods

### Study subjects

This study was conducted in accordance with a protocol (reference number: 20-000372) reviewed and approved by the Mayo Clinic Institutional Review Board. Confidentiality was maintained for study participants.

### Generation of iPSC

We used whole blood in EDTA tubes to isolate peripheral blood mononuclear cells (PBMCs) from subjects with OUD, and PBMCs for iPSC reprogramming using the CytoTune™-iPS 2.0 Sendai Reprogramming Kit (A16517, Thermo Fisher, USA). We then characterized OUD patient-derived iPSCs as previously described [14, 15]. Briefly, iPSCs were cultured on matrigel-coated plates (BD Biosciences) in mTeSR1 Plus medium (STEMCELL technology, MA, USA). All iPSC lines revealed normal karyotypes. They all expressed pluripotency markers, and they were regularly verified to be free from mycoplasma.

### Generation of iPSC-derived forebrain organoids

We generated 3-D iPSC-derived forebrain organoids. Briefly, pre-patterned floating embryonic bodies (EBs) formed from intact iPSC colonies were embedded in matrigel and cultured with 1x N2, 1x NEAA and 1x Glutamax (Invitrogen, Grand Island, NY), 1 μM SB431542, and 1 μM CHIR99021 (Selleckchem, Carlsbad, CA) for 7 days. On day 14, organoids were mechanically dissociated from the matrigel and cultured in a bioreactor [16]. Culture medium from days 14–70 consisted of DMEM/F12 medium supplemented with 1x N2, 1x B27, 1x NEAA and 1x Glutamax, 1× 2-metabptoethanol, 100x penicillin-streptomycin solution, and 2.5 μg/ml insulin (Sigma-Aldrich, St Louis, MO). The medium was changed every other day. From day 70 onward, supplementing media with 20 ng/ml BDNF, 20 ng/ml GDNF (Peprotech, Rocky Hill, NJ), 0.2 mM L-Ascorbic Acid, and 0.5 mM cAMP (Sigma-Aldrich, St Louis, MO) was used [17].

### Generation of iPSC-derived neurons and astrocytes

As previously described, we differentiated the iPSCs into neurons [18]. Briefly, iPS cells were cultured on Matrigel with mTeSR1 Plus media (STEMCELL technology, MA, USA). We suspended 3-D iPSC aggregates in embryoid body (EB) medium, consisting of FGF-2-free iPS cell medium supplemented with 2 μM dorsomorphin (sigma) and 2 μM A-83 (sigma), in non-treated polystyrene plates for 6 days with a daily medium change. After 6 days, we replaced EB medium by neural induction medium (NPC medium) consisting of DMEM/F12, 1x N2 supplement, 1x NEAA, 2 ug ml^−1^ heparin (Tocris Bioscience) and 2 μM cyclopamine (Tocris Bioscience). The floating EBs were then transferred to Matrigel-coated plates at day 7 to form neural tube-like rosettes. The attached rosettes were kept for 15 days with NPC medium change every other day. On day 22, we transferred the rosettes to low attachment plates in NPC medium containing 1x B27 (Thermo fisher). After two days in culture, for astrocyte differentiation, resuspended neural progenitor spheres were dissociated with Accutase (STEMCELL technology, MA, USA) and placed onto Matrigel-coated plates in astrocyte medium (ScienCell, cat#1801). Neuronal culture medium consisted of Neurobasal medium supplemented with 1x glutamax, 1x B27, 0.2 mM L-Ascorbic Acid, and 0.5 mM cAMP (Sigma-Aldrich, St Louis, MO), 10 ng ml^−1^ BDNF and 10 ng ml^−1^ GDNF. Medium was replaced every three days during continuous culture [18]. Most iPSC-derived neurons (>90%) are glutamatergic excitatory neurons that express α-CAMKII and VGLUT1 (Fig. S1). The iPSC-derived neurons functionally mature four weeks after differentiation from NPCs. For example, they form synapses, fire action potentials, and have spontaneous synaptic activity [18]. Neurotransmitters i.e. serotonin, GABA, and epinephrine, were also detected in the iPSC-derived neurons [19].

### Immunofluorescence staining and confocal imaging analysis

Cells were fixed in 4% paraformaldehyde at room temperature for 15 min. Cells were permeabilized with 0.2% Triton X-100 in PBS. After blocking, cells were incubated with the primary antibody in 5% BSA (see Table S1) overnight. The secondary antibody (1:2000 dilution) was then used. Antifade mounting media with dapi (VECTOR laboratory, Burlingame, CA, USA) was used to stain the nuclei. Fluorescence microscopy (Olympus, FV1200) was used to visualize slides.

### Drug treatment

Drug treatment was conducted at 83–90 days of forebrain organoid differentiation and at 28–34 days of forebrain neuron differentiation. The concentrations of oxycodone (50 µg/L) [20] and buprenorphine (2 ng/mL) [21, 22] used to perform these experiments were selected to fall within the range of blood drug concentrations in patients taking standard clinical doses of these two drugs. 3-D iPSC-derived brain organoids were cultured in the bioreactors with the drugs for seven days, and the medium was changed daily. Fludarabine (S1491, Selleckchem, USA) is a STAT1 inhibitor that causes a specific depletion of STAT1 protein (and mRNA) but not of other STATs. This compound was used to treat iPSC-derived neurons for 24 h, and cells were collected for RNA isolation.

### Single nuclei RNA sequencing (snRNA-seq) and data analysis

We isolated nuclei from frozen iPSC-derived forebrain organoids and constructed 3’ single cell gene expression libraries (Next GEM v3.1) using the 10x Genomics Chromium system. Each library was sequenced with Illumina NovaSeq (PE150) as summarized in Fig. S2. snRNA-seq libraries were sequenced using a HiSeq4000 at Singulomics (New York, NY, USA). After sequencing, clean reads were analyzed with human reference genome GRCh38-2020-A using Cell Ranger v6.0.1 software. Output from Cell Ranger in the form of gene-barcode matrix were analyzed using R package Seurat version 3.2.3 [23–25]. Genes were filtered to remove those expressing in less than three cells. Cells were also filtered to only keep those with at least 500 expressed genes, at least 1000 unique molecular identifiers (UMIs) and mitochondrial gene content less than 50% UMI. Cells with UMI counts larger than the average UMI of all cells plus three times the standard deviation were further removed. Raw counts were natural log-transformed and multiplied by a factor of 1000. A subset of 2000 genes with highest variance across all the cells were selected as variable features. The dataset was then centered and scaled after regressing out the number of UMI of each cell. Principal component analysis (PCA) was performed on the previously identified 2000 most variable genes and an elbow plot showing number of PCs vs. cumulative total variance explained was generated to decide the optimal number of top principal components (PCs) to use for downstream analysis, which was 10 in this study. The Uniform Manifold Approximation and Projection (UMAP) method was used to project the cells represented by 10 PCs into two-dimensional scatter plots for visualization. A graph-based clustering approach was used to group cells into clusters. First, a k-nearest neighbor (KNN) graph was constructed based on the selected top PCs using euclidean distance. Pairwise edge weights of cells were refined based on the shared overlap in their local neighborhoods (Jaccard similarity). This step was achieved by calling function FindNeighbors in the Seurat package. A modularity optimization process using the Louvain algorithm was applied to iteratively group cells together, with the goal of optimizing the standard modularity function. This is implemented in the function FindClusters in the Seurat package. A resolution of 0.6 was used to control the granularity of the clusters. Cluster marker genes were determined using function FindAllMarkers for each cluster and filtered by Bonferroni-corrected *p* value <0.05 and log2 fold change >0.25, and expressing in at least 25% of cells in the cluster. Cluster marker genes were compared to published cell type markers to identify cell types. Differential expression measures were retained as significant when adjusted p values were below a false discovery rate (FDR) cut-off of 0.05 [26, 27]. The Search Tool for the Retrieval of Interacting Genes (STRING; http://string-db.org; version 11.5) was used to construct the protein-protein interaction network [28].

### Real time PCR

The PCR reactions contained total RNA (100 ng), 5 µl of 2X SYBR green qPCR master mix, 0.08 µl of DNA polymerase, 1 µl of gene specific primer (Table S1) and distilled water up to 10 µl final volume per reaction. Real time PCR reactions were performed in duplicate using the Applied Biosystems QuantStudio 5 Real-Time PCR System (Life Technologies, Carlsbad, CA, USA). The 2^−ΔΔCt^ method was employed for statistical data analysis.

### Western blot analysis

Protein samples were isolated from iPSC-derived neurons. Protein samples were loaded into the wells of the SDS-PAGE gel. proteins were transferred onto PVDF membranes. After blocking, PVDF membranes were incubated with primary antibodies at 4 °C overnight (Table S1). Then, the washed membranes were incubated with secondary antibody (1:2000) for an hour at room temperature. The membranes were incubated with ECL substrate (Thermo Scientific, Madison, WI, USA) for 3 min and were visualized using the geldoc go imaging system (Bio-rad, USA).

### Statistical analysis

We performed statistical analysis using R Statistical Software (version 4.0.6; R Foundation for Statistical Computing, Vienna, Austria). ELISA data were analyzed using ANOVA, followed by Tukey’s multiple comparison tests for individual comparisons when significant effects were detected. *P* < 0.05 was considered statistically significant.

## Results

### Gene expression profiles in iPSC-derived brain organoids

**Fig. 1:**
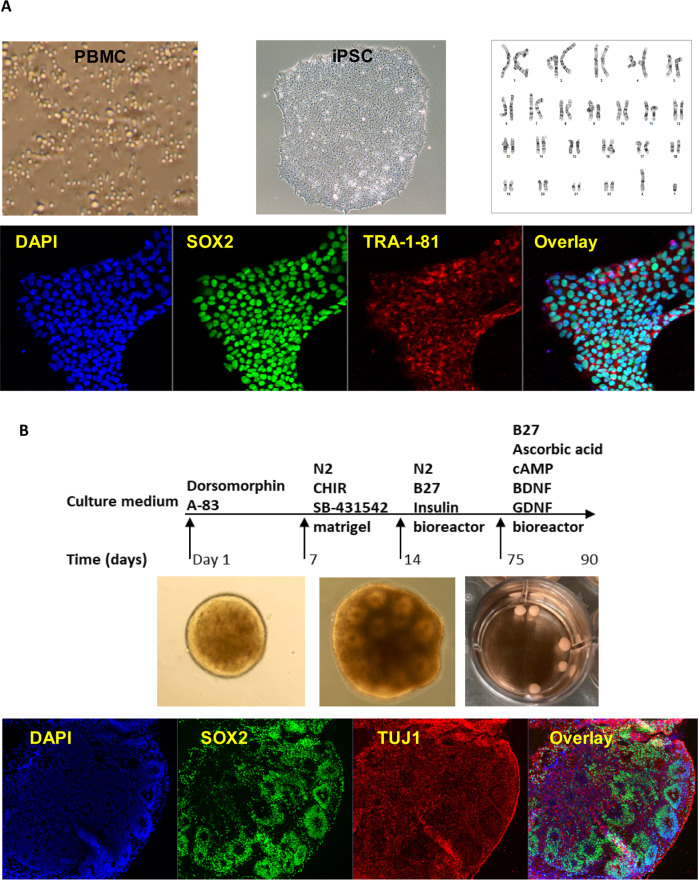
Generation of iPSC-derived forebrain organoids. **A** iPSC generation using peripheral blood mononuclear cells (PBMC). All iPSC cell lines displayed normal karyotypes and were positive for pluripotency markers. The panel below the schematic displays representative examples of staining for iPSC pluripotency markers: SOX2, and TRA-1-81. **B** A schematic outline of procedures used during the differentiation of iPSC-derived forebrain organoids. The panel below the schematic displays representative examples of staining for SOX2 and TUJ1.

**Fig. 2:**
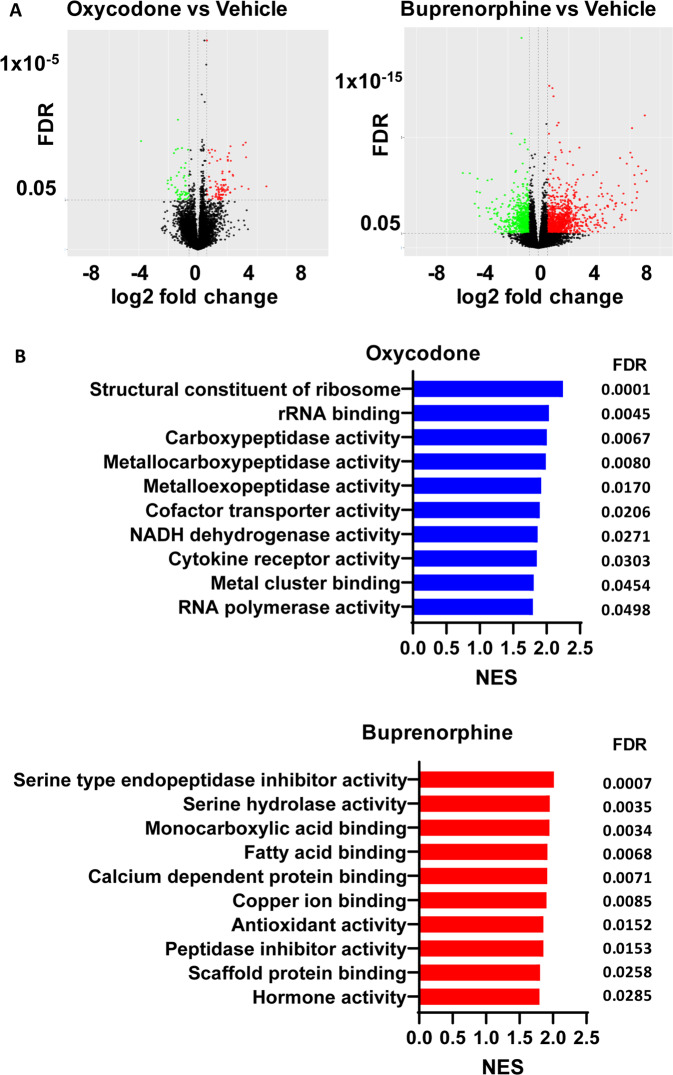
Bulk RNA-seq using iPSC-derived brain organoids from three male patients with OUD. **A** Volcano plots indicate differentially expressed genes with FDR 0.05. **B** Pathway analysis of the bulk RNA-seq data was performed using gene set enrichment analysis (GSEA) software [43, 44]. NES is the normalized enrichment score to account for the size of each gene set.

Forebrain organoids were generated using iPSCs from three male subjects with OUD (Fig. 1). We set out to explore the effects of oxycodone and buprenorphine on iPSC-derived forebrain organoids. Specifically, we generated iPSC-derived forebrain organoids, which expressed all three subtypes of opioid receptors (Fig. S2), and treated them with vehicle, oxycodone (opioid receptor agonist) or buprenorphine (partial opioid receptor agonist) for seven days. The concentrations of oxycodone (50 µg/L) [20] and buprenorphine (2 ng/mL) [21, 22] used to perform these experiments were selected to fall within the range of blood drug concentrations in patients taking standard clinical doses of these two drugs. We performed bulk RNA-seq and identified 279 and 3333 genes for which expression was significantly altered (FDR < 0.05) after exposure to oxycodone or buprenorphine treatment, respectively, as compared to vehicle treatment (Fig. 2A). We then performed pathway analysis for each drug treatment condition (Fig. 2B). The most common and most highly affected pathways in the presence of oxycodone differed from buprenorphine. However, bulk-RNA-seq might not be able to provide detailed insight into the mechanism of action of these drugs. Therefore, we performed single-cell sequencing using the same batch of iPSC-derived forebrain organoids.

### Single-cell sequencing and iPSC-derived forebrain organoids

**Fig. 3:**
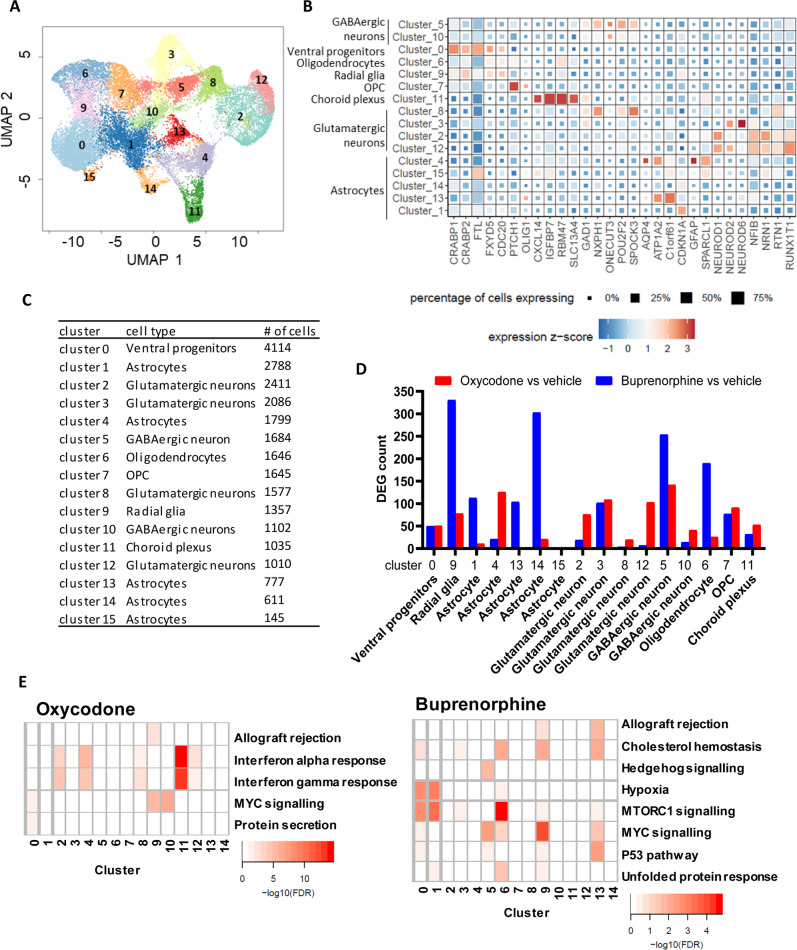
snRNA-seq using iPSC-derived brain organoids from three male patients with OUD. **A** UMAP plot of snRNA-seq data, which included 25787 nuclei. **B** Heatmap of canonical genes to classify UMAP clusters 0–15 from Fig. 3A. Cluster identifiers are labeled on the y axis and canonical marker genes are labelled on the x axis. Darker shades of red and size of the squares represent greater expression of genes and percentage of cell expressing the gene. **C** The number of cells in each cluster identified. **D** The number of differentially expressed genes (DEG) in each cluster in response to oxycodone and buprenorphine. **E** Pathway analysis was performed using the results derived from the differential gene expression analysis in each cell cluster.

We analyzed 25,787 nuclei using snRNA-seq, including 9151cells from organoids treated with vehicle, 7623 cells from oxycodone treated organoids, and 9013 cells from buprenorphine treated organoids after quality control filtering (Fig. 3A, and Fig S2D–F). The single-cell sequencing data were visualized using uniform manifold approximation and projection (UMAP) and revealed 16 transcriptionally distinct clusters (Fig. 3A) containing treated and untreated organoids from three subjects. We then annotated these clusters using known cellular markers of major brain cell types (Fig. 3B, C, Fig. S3 and Table S2) [29, 30].

As a first step, we set out to determine the cell-type specific and drug-specific gene expression profiles in iPSC-derived forebrain organoids. Differential gene expression analysis was performed for each cluster to determine the effect of the drugs on iPSC-derived forebrain organoids in a cell-type specific fashion. Our results showed that oxycodone and buprenorphine displayed distinct gene expression profiles. Specifically, oxycodone affected transcriptional response primarily in neurons, whereas buprenorphine significantly influenced transcription regulation in glial cells (Fig. 3D). Pathway analysis showed that oxycodone induced the type I interferon signaling pathway in many cell types, including neurons and astrocytes, whereas the mTOR signaling pathway was the most commonly affected pathway in response to buprenorphine treatment (Fig. 3E and Table S3 for complete results). Similar results were observed when we performed differential gene expression after pseudo-bulking by grouping clusters based on the cell types. Of importance, the mTOR signaling pathway was the most significant pathway which was associated with genes that altered their expression in astrocytes in response to buprenorphine treatment (FDR: 5.47E-05), while the type I interferon signaling pathway was the most significant pathway associated with genes that altered their expression in neurons in response to oxycodone treatment (FDR: 0.07). As a result, iPSC-derived neurons were included in the subsequent functional genomic studies.

### Oxycodone induced the type I interferon signalling pathway

**Fig. 4:**
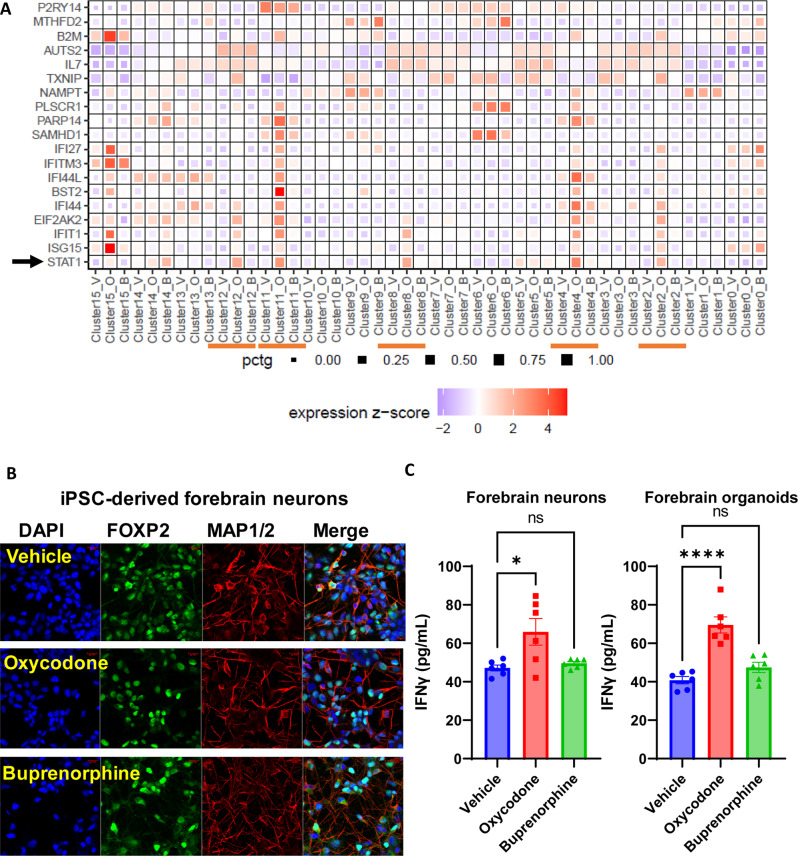
Interferon signalling pathway was affected by oxycodone but not by buprenorphine. **A** the heatmap demonstrates that mRNA expression of the selected genes involved in interferon signalling pathways were mostly upregulated in clusters 2, 4, 8, 11, and 12 in response to oxycodone. V vehicle, O oxycodone, B buprenorphine. selected genes involved in interferon signalling pathways are labeled on the y axis and treatment conditions in each cell cluster are labelled on the x axis. Darker shades of red and size of the squares represent greater expression of genes and percentage of cell expressing the gene. **B** representative immunostaining of iPSC-derived forebrain neurons in response to oxycodone and buprenorphine. **C** IFNγ concentrations were quantified using ELISA in iPSC-derived forebrain neurons (*F*(2,15) = 6.067, p:0.01) and forebrain organoids (*F*2,15) = 24.18, *p* < 0.0001.

**Fig. 5:**
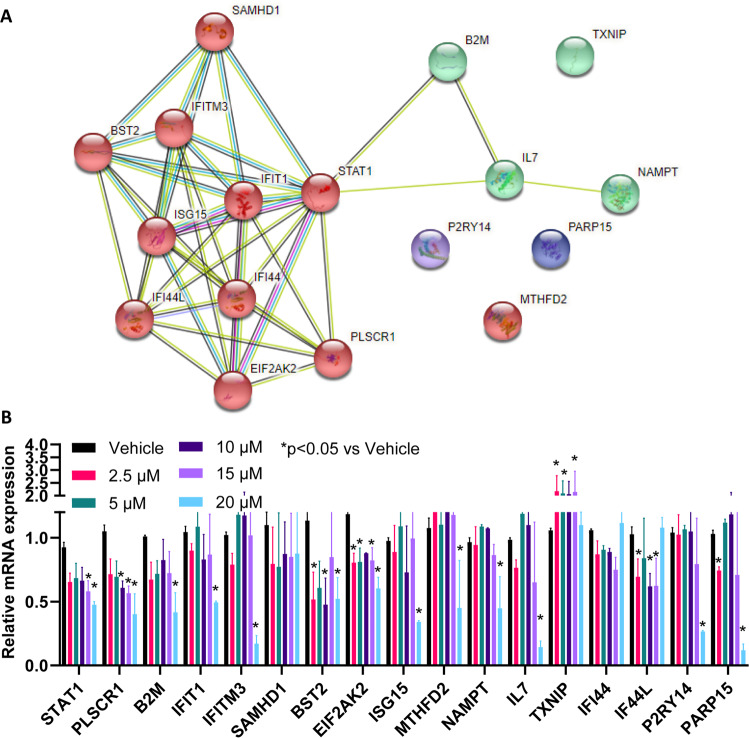
STAT1 can regulate a large number of inflammatory mediators in interferon signaling pathways. **A** protein-protein interaction network of genes shown in Fig. 4A was constructed by using the STRING database (https://string-db.org/). The number of lines indicates the strength of predicted functional interactions between the proteins using a Markov Cluster Algorithm. The color of the lines connecting the nodes indicates the particular lines of evidence used to establish a functional association. There is no particular meaning of the node color itself because they are used only as a visual aid. The distance between the nodes is a measure of the confidence of the interaction as determined by a Bayesian scoring system. **B** iPSC-derived neurons (*n* = 3) were used to determine relative mRNA expression of genes shown in Fig. 4A in response to STAT1 inhibitor that ranged from 2.5 µM to 20 µM.

**Fig. 6:**
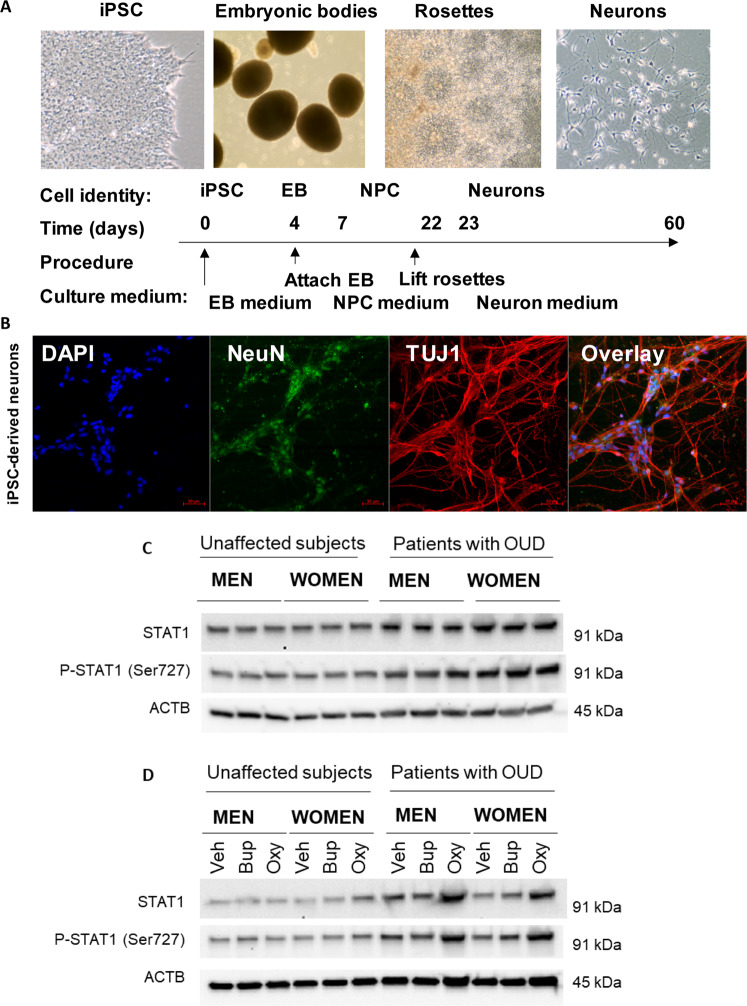
STAT1 expression was significantly induced by oxycodone in patients with OUD. **A** A schematic outline of procedures used during the differentiation of iPSC-derived forebrain neurons. **B** Representative examples of staining for neuronal markers: NeuN and TUJ1. **C** The basal level of protein expression of STAT1 was determined using the iPSC-derived neurons from male and female OUD patients and unaffected controls (*n* = 3 each group). STAT1 protein expression was significantly higher in OUD patients as compared to unaffected controls. **D** Representative Western blot images demonstrate that STAT1 expression went up in response to oxycodone treatment in iPSC-derived neurons from patients with OUD. However, STAT1 protein expression in unaffected controls displayed no difference in response to drug treatment. In parallel, p-STAT1 (Ser727) was upregulated in response to oxycodone treatment in iPSC-derived neurons from patients with OUD. veh vehicle, bup buprenorphine, oxy oxycodone.

The heatmap (Fig. 4A) demonstrated gene expression as determined by snRNA-seq for selected genes in the type I interferon signaling pathway. In clusters 2, 4, 8, 11, 12, most genes in this pathway appeared to have higher gene expression in the presence of oxycodone than vehicle. In contrast, for the same genes, the expression displayed no difference between buprenorphine and vehicle treatment (Fig. 4A). Several clusters, including clusters 2, 8, and 12, were neurons. As a result, we measured interferon-gamma (IFNγ) concentrations in both iPSC-derived neurons and brain organoids before and after the cells were exposed to oxycodone or buprenorphine (Fig. 4B). Strikingly, IFNγ concentrations were induced by oxycodone (Fig. 4C). However, buprenorphine had no effect on IFNγ concentrations in iPSC-derived forebrain neurons. Similar results were observed in iPSC-derived forebrain organoids (Fig. 4C). We next constructed a protein-protein interaction network using the differentially expressed genes in the interferon signaling pathways, as shown in Fig. 4A. Among the genes listed in Fig. 4A, STAT1 is a transcription factor that appears to interact with several genes in the interferon signaling pathway, which could be activated by oxycodone (Fig. 5A). We subsequently cultured iPSC-derived neurons and exposed them to fludarabine, a STAT1 inhibitor that causes a specific deletion of STAT1 but not other STATs. Of importance, a series of genes involved in the interferon signaling pathway, as shown graphically in Fig. 5A altered their mRNA expression in response to fludarabine treatment in a dose-dependent fashion (Fig. 5B). These results suggest that STAT1 could regulate a series of genes in the interferon signaling pathway. Other research confirms that *STAT1* is one of the master transcription factors that can regulate a large number of inflammatory mediators, including those in interferon signaling pathways in patients with OUD compared to unaffected controls [31, 32]. In line with our snRNA-seq results, STAT1 was induced by oxycodone in iPSC-derived brain organoids in patients with OUD (Fig. 4A). We then extended this observation by including iPSC-derived neurons from OUD subjects and unaffected controls (Fig. 6A, B). Strikingly, our results showed that, the basal level of STAT1 expression was significantly higher in patients with OUD as compared with unaffected controls (Fig. 6C) and, even more striking, that STAT1 expression was significantly induced by oxycodone only in OUD patients (Fig. 6D). Furthermore, phospho-STAT1 (Ser727) were upregulated in response to oxycodone treatment in patients with OUD. However, buprenorphine did not affect those genes (Fig. 6D). We did not observe sex-differences in effect on STAT1 protein expression (Fig. 6D). These results suggest that upregulation of STAT1 might be associated with OUD, and that STAT1 might play a role in transcriptional regulation in response to oxycodone. We took one step futher and generated iPSC-derived astrocytes to confirm that oxycodone induced STAT1 protein expression might be cell-type specific (Fig. S4). Sepcifically, there is no significant difference for the basal level of STAT1 protein expression in iPSC-derived astrocyte between patients with OUD and unaffected controls (Fig. S4C). This set of experiments also demonstrates that oxycodone was not able to induce STAT1 protein expression in iPSC-derived astrocytes from either patients with OUD or unaffected controls (Fig. S4D). Taken together, this series of studies demonstrated that the systematic experimental approach that we had taken might represent a potentially important step toward generating hypotheses that could lead to a deeper understanding of the molecular mechanism for disease pathogenesis and drug action, as well as opening new avenues for the discovery of novel therapeutic targets for the treatment of OUD.

## Discussion

The opioid epidemic represents a national crisis. The number of opioid prescriptions of oxycodone, fentanyl, and morphine, continues to be a significant public health concern [33]. Oxycodone is one of the most prescribed opioid medications in the United States. For example, the Mayo Clinic Right 10K study (*n* = 11,098) is an institutional resource that has recently reported that over 50% of subjects have received at least one prescription for oxycodone during 2005–2017 [34, 35]. In addition, over 4000 subjects were prescribed two or more opioids [34, 35]. Oxycodone is a full opioid receptor agonist, whereas buprenorphine is a partial opioid agonist. The opioid substitution treatments that have been used clinically include two main medications: buprenorphine and methadone [36]. Buprenorphine, a partial agonist at the mu-opioid receptor and an antagonist at the kappa opioid receptor, is currently the most prescribed medication for OUD pharmacotherapy [12, 13]. Therefore, we set out to study how these drugs might modulate brain cell types at the single-cell level. The present study represent**s** a systematic attempt to study drug mechanism(s) of action and the underlying pathophysiology responsible for opioid addiction.

We hypothesized that oxycodone and buprenorphine might regulate somewhat similar biological pathways in iPSC-derived brain organoids from patients with OUD. We began with bulk RNA-seq using iPSC-derived brain organoids to test that hypothesis and observed that oxycodone and buprenorphine displayed surprisingly distinct gene expression profiles (Fig. 2). However, bulk RNA-seq cannot provide detailed insight into the molecular mechanism of drug action, i.e., specific cell type alternation. As a result, we took a step further by performing single-cell transcriptomics using the same samples. Those studies identified a new layer of molecular regulation associated with OUD that might potentially open new avenues for future drug development to treat and/or prevent substance use disorders. As expected, we observed that buprenorphine and oxycodone modulated distinct gene sets and biological pathways at the single cell level (Fig. 3). These results imply that different opioids do not share the same biological effects or mechanisms [37].

Our results suggest that buprenorphine significantly influences transcriptional regulation in glial cells. However, oxycodone induced type I interferon signaling in many cell types, including neural cells in brain organoids (Fig. 4). Functional genomics studies demonstrated that oxycodone could activate STAT1, influencing transcription regulation in the interferon signaling pathway (Fig. 5). It has been reported that oxycodone self-administration exposure altered numerous genes related to immune function in the dorsal striatum and ventral striatum in mice [38]. Several preclinical studies have reported brain cell-type and drug-specific responses using single-cell sequencing. Specifically, a previous study used single-cell RNA-seq to uncover cell type-specific responses to morphine in the brains of mice and observed that genes affected by morphine were enriched in biological pathways linked to oligodendrocyte maturation and myelination [39]. In addition, a recent study has reported single-cell resolution of cocaine-induced transcriptional regulation and identified specific medium spiny neuron subpopulations in rats that altered neuronal functional and behavioral responses to cocaine [40]. These observations suggest that each opioid agent might have unique molecular profiles and mechanisms of action and highlight the need for more standardized models to evaluate drug action in the brain at the single-cell level.

Our study was designed to apply a systematic genome-wide approach to identify gene expression profiles in iPSC-derived brain organoids before and after drug exposure at single-cell resolution. These “big data” from OUD patients have allowed us to generate novel hypotheses and to examine molecular mechanisms associated with OUD and response to OUD pharmacotherapy. However, iPSC-derived brain organoids, which can recapitulate important brain architecture, have limitations. For example, iPSC-derived brain organoids are region-specific. Forebrain organoids were selected because previous preclinical and clinical studies identified a key involvement of the prefrontal cortex in addiction [41, 42]. In addition, each drug studied only included one concentration at one particular time point during the differentiation of iPSC-derived forebrain organoids. We speculate that effects may scale with increasing doses and/or longer duration of exposure. Future studies that include different brain regions will be required to determine the brain-region specific effects of response to oxycodone and buprenorphine. Our single cell sequencing was performed using iPSC-derived brain organoids from three male subjects with OUD, therefore, our study was not able to explore possible sex-difference effects at single-cell level. However, despite these limitations, our work still represents an important contribution by providing novel mechanistic insight into the drug action of two commonly used opioids, oxycodone, and buprenorphine, at single-cell resolution.

In summary, the present study utilized iPSC-derived forebrain organoids and single-cell sequencing technology as unbiased tools to study cell-type-specific and drug-specific transcriptional responses. Our results revealed distinct transcriptional responses to oxycodone and buprenorphine by iPSC-derived brain organoids from patients with OUD. Oxycodone activated STAT1 and induced type I interferon signaling in patients with OUD. Finally, we demonstrated that elevation of STAT1 expression associated with OUD might have a role in transcriptional regulation in response to oxycodone but not buprenorphine. These results may provide novel mechanistic insight into drug action at single-cell resolution.

## Supplementary information

Supplementary Figures

Supplementary Figure and Table legend

Supplementary Tables

## Data Availability

All data supporting our findings can be found in the main paper or in supplementary files. Sequencing data are available via the GEO accession number: GSE210206.
