# Supplementary material for: Single cell transcriptomics reveals distinct transcriptional responses to oxycodone and buprenorphine by iPSC-derived brain organoids from patients with opioid use disorder
Source: Mol Psychiatry. 2022 Oct 27;29(6):1636–46. doi: 10.1038/s41380-022-01837-8 (PMC10588459; doi:10.1038/s41380-022-01837-8)
Supplement: Supplementary file 1 — Supplementary Figures [file 41380_2022_1837_MOESM1_ESM.pdf]

Supplementary materials

Single cell transcriptomics reveals distinct transcriptional responses to oxycodone and buprenorphine by iPSC-derived brain organoids from patients with opioid use disorder

Ming-Fen Ho, Ph.D.[1,2*], Cheng Zhang, Ph.D.[2], Irene Moon, M.S[2], Xiujuan Zhu, Ph.D.[2], Brandon J. Coombes, Ph.D.[3], Joanna Biernacka, Ph.D.[3], Michelle Skime, M.S.[1], Tyler S. Oesterle, MD. M.P.H.[1], Victor M Karpyak, MD, Ph.D.[1], Kristen Schmidt, M.D.[4], Kate Gliske, Ph.D.[4], Quyen Ngo, Ph.D.[4], Cedric Skillon, M.D.[4], Marvin D. Seppala, M.D.[4] Hu Li, Ph.D.[2], and Richard M. Weinshilboum, M.D.[2*]

Fig. S1 A schematic outline of procedures used during the differentiation of iPSC-derived forebrain neurons. (B) Representative examples of staining for neuronal markers. Most iPSC-derived neurons (>90%) are glutamatergic excitatory neurons that express vesicular Glutamate Transporter 1 (VGLUT1). Approximately 5-10 % neurons are GABAergic neurons that express glutamate decarboxylase 1 (GAD1), and less than 1% neurons are dopaminergic neurons that express tyrosine hydroxylase (TH).

Fig. S2 (A) mRNA expression of three subtypes of opioid receptor (OPRM1, OPRK1, and OPRD1) in iPSC-derived forebrain organoids. (B) demographic and characteristics of the three male subjects included in the snRNA-seq study and cell lines for functional genomics studies. (C) bulk RNA-seq matrix of iPSC-derived forebrain organoids (n=3). (D) snRNA-seq matrix of iPSC-derived forebrain organoids. (E) UMAP plot demonstrates the distributions of cell in each treatment condition (V: vehicle, O: oxycodone, B: buprenorphine). (F) Percentage of cells from each subject and each treatment condition which were used to generate the UMAP plot as shown in Supplementary figure 2E.

Fig. S3 UMAP of cells colored by canonical marker genes list in supplementary table 2.

Fig. S4 (A) A schematic outline of procedures used during the differentiation of iPSC-derived forebrain astrocytes. (B) Representative examples of staining for astrocyte markers: S100B and GFAP. (C) Protein expression of STAT1 was determined using the iPSC-derived astrocyte from male and female OUD patients and healthy controls (n=3 each group). The basal level of STAT1 protein expression showed no difference between OUD and unaffected controls. (D) Representative Western blot images demonstrate that STAT1 expression did not alter in response to oxycodone or buprenorphine treatment in iPSC-derived astrocytes from patients with OUD or unaffected controls. veh: vehicle, bup: buprenorphine, oxy: oxycodone.

Fig. S1

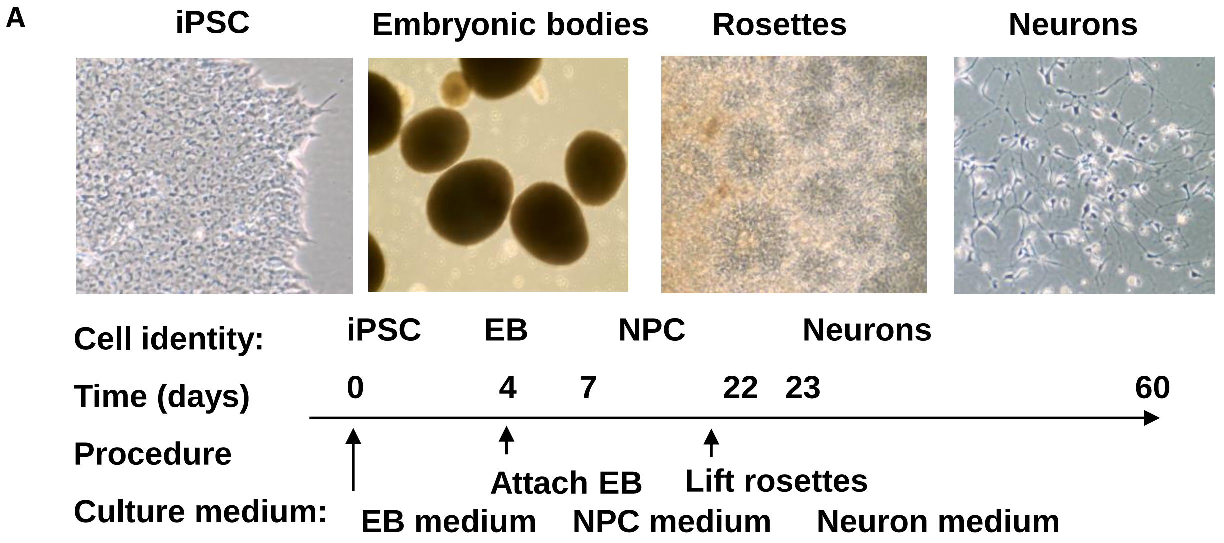
A
iPSC
Embryonic bodies
Rosettes
Neurons
Cell identity:
iPSC
EB
NPC
Neurons
Time (days)
0
4
7
22
23
60
Procedure
Attach EB
Lift rosettes
Culture medium:
EB medium
NPC medium
Neuron medium

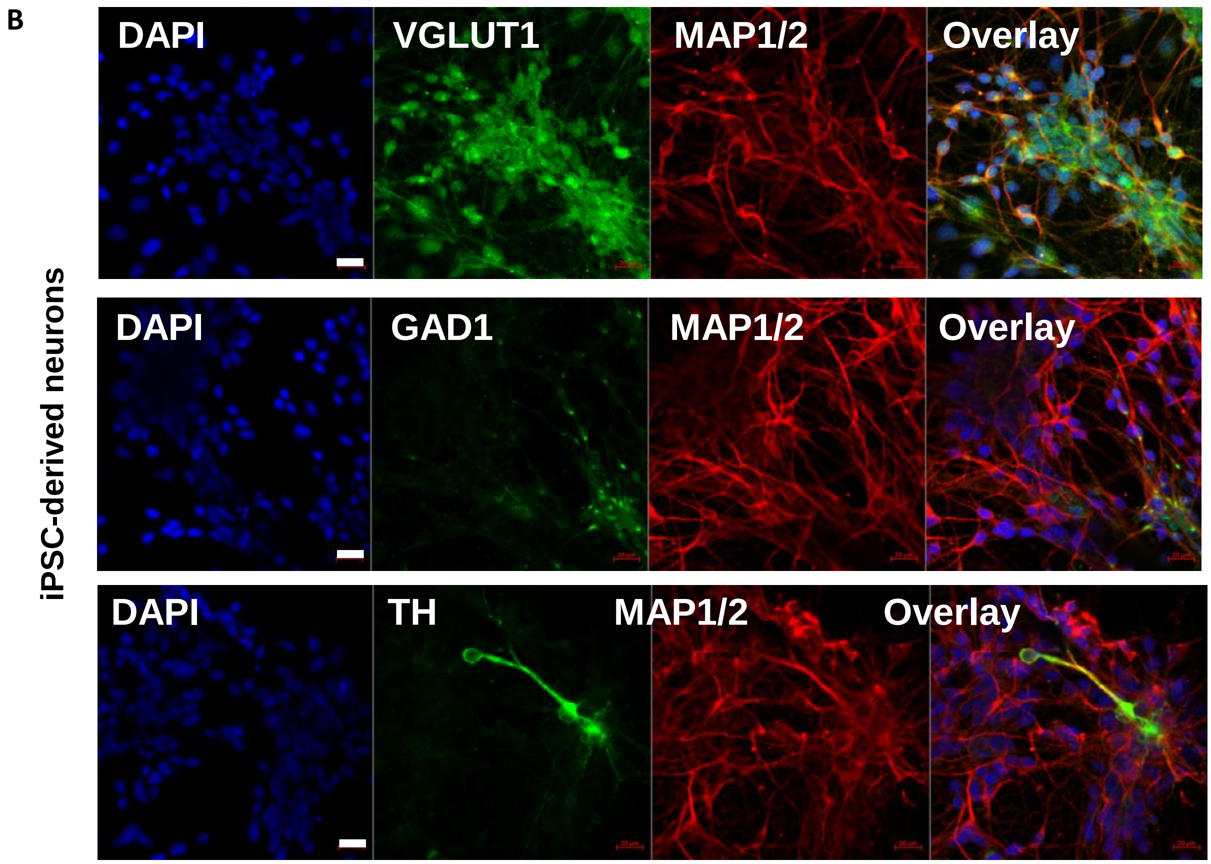
B
iPSC-derived neurons
DAPI
VGLUT1
MAP1/2
Overlay
DAPI
GAD1
MAP1/2
Overlay
DAPI
TH
MAP1/2
Overlay

Fig. S2

A

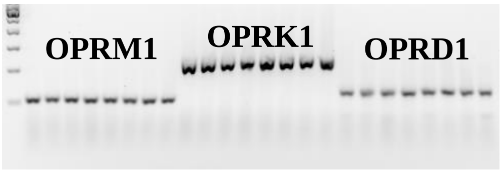

B **Demographic and characteristics of the subjects**

OUD			Unaffected controls		
subject	sex	age	subject	sex	age
1003	M	29	HCM03	M	46
1004	M	40	HCM07	M	43
1005	M	33	HCM09	M	31
1028	F	58	HC008	F	58
1030	F	26	HC010	F	26
1038	F	19	HC011	F	19

C **Bulk RNA-seq matrix**

Sample ID	# Reads	Yield (Mbases)	Mean Quality Score	% Bases >= 30
OFO1003B1	22192146	6658	35.52	91.96
OFO1003B2	21721770	6517	35.52	91.92
OFO1003O1	18244006	5473	35.59	92.27
OFO1003O2	19828525	5949	35.59	92.3
OFO1003V1	24192866	7258	35.55	92
OFO1003V2	23830631	7149	35.57	92.12
OFO1004B1	21777941	6533	35.7	92.69
OFO1004B2	21692316	6508	35.58	92.23
OFO1004O1	20157507	6047	35.36	91.19
OFO1004O2	24663388	7399	35.64	92.45
OFO1004V1	22025057	6608	35.5	91.77
OFO1004V2	16284365	4885	35.63	92.41
OFO1005B1	26196093	7859	35.69	92.61
OFO1005B2	24090712	7227	35.66	92.57
OFO1005O1	17651174	5295	35.71	92.75
OFO1005O2	19293992	5788	35.64	92.46
OFO1005V1	23177285	6953	35.65	92.47
OFO1005V2	18348190	5504	35.67	92.58

D **snRNA-seq**

Sample	Raw reads	Raw data(G)	Effective(%)	Error(%)	Q20(%)	Q30(%)	GC(%)
FO1003V	1028124614	154.2	99.99	0.07	86.59	75.96	39.88
FO1003B	912763852	136.9	99.99	0.07	86.74	76.31	40.51
FO1003O	1025035660	153.8	99.99	0.07	86.56	75.93	39.95
FO1004V	1038383120	155.8	99.99	0.07	86.92	76.94	41.70
FO1004B	1116750966	167.5	99.99	0.07	86.94	76.98	42.52
FO1004O	974060194	146.1	99.99	0.07	86.63	76.62	42.02
FO1005V	919346794	137.9	100.00	0.07	87.43	79.25	40.48
FO1005B	902854586	135.4	100.00	0.06	87.47	79.34	41.27
FO1005O	955474068	143.3	100.00	0.06	87.52	79.50	41.16

E

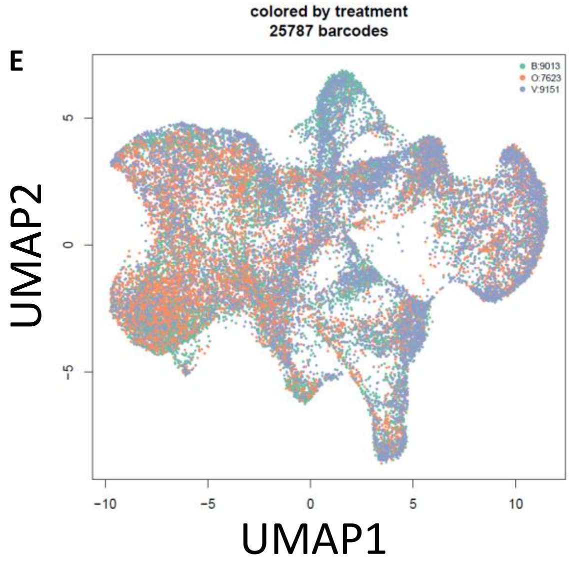

F

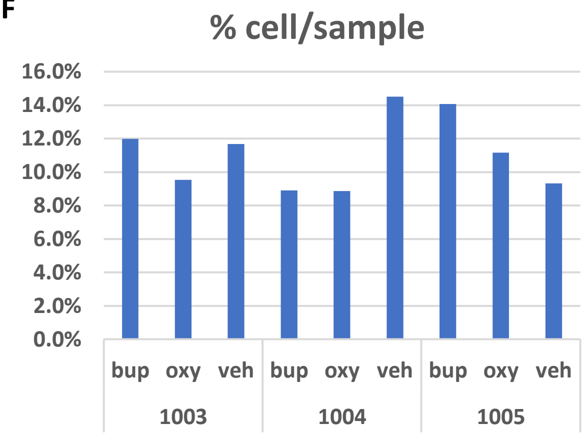

Fig. S3

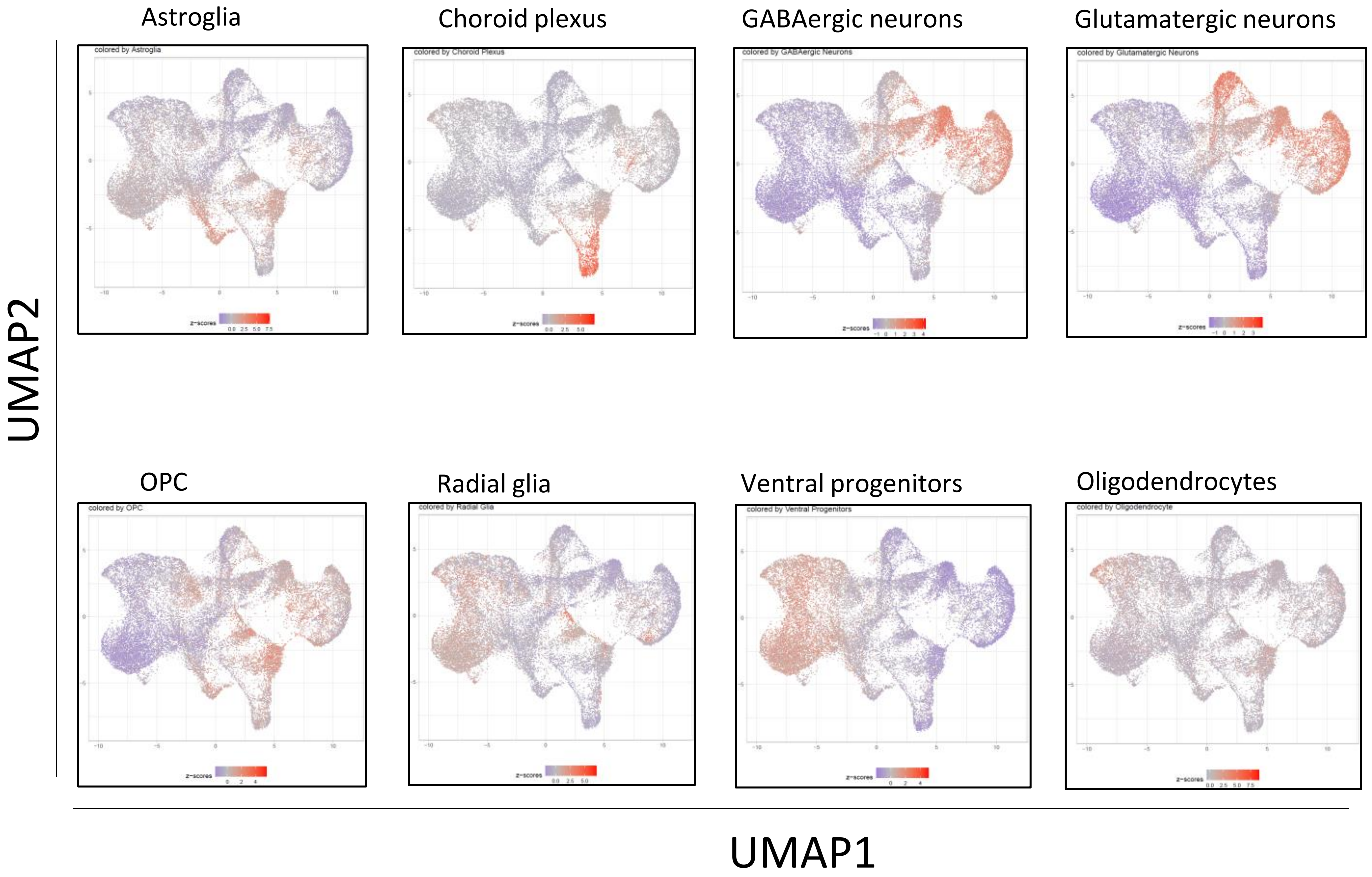

Astroglia
Choroid plexus
GABAergic neurons
Glutamatergic neurons
OPC
Radial glia
Ventral progenitors
Oligodendrocytes
UMAP2
UMAP1

Fig. S4

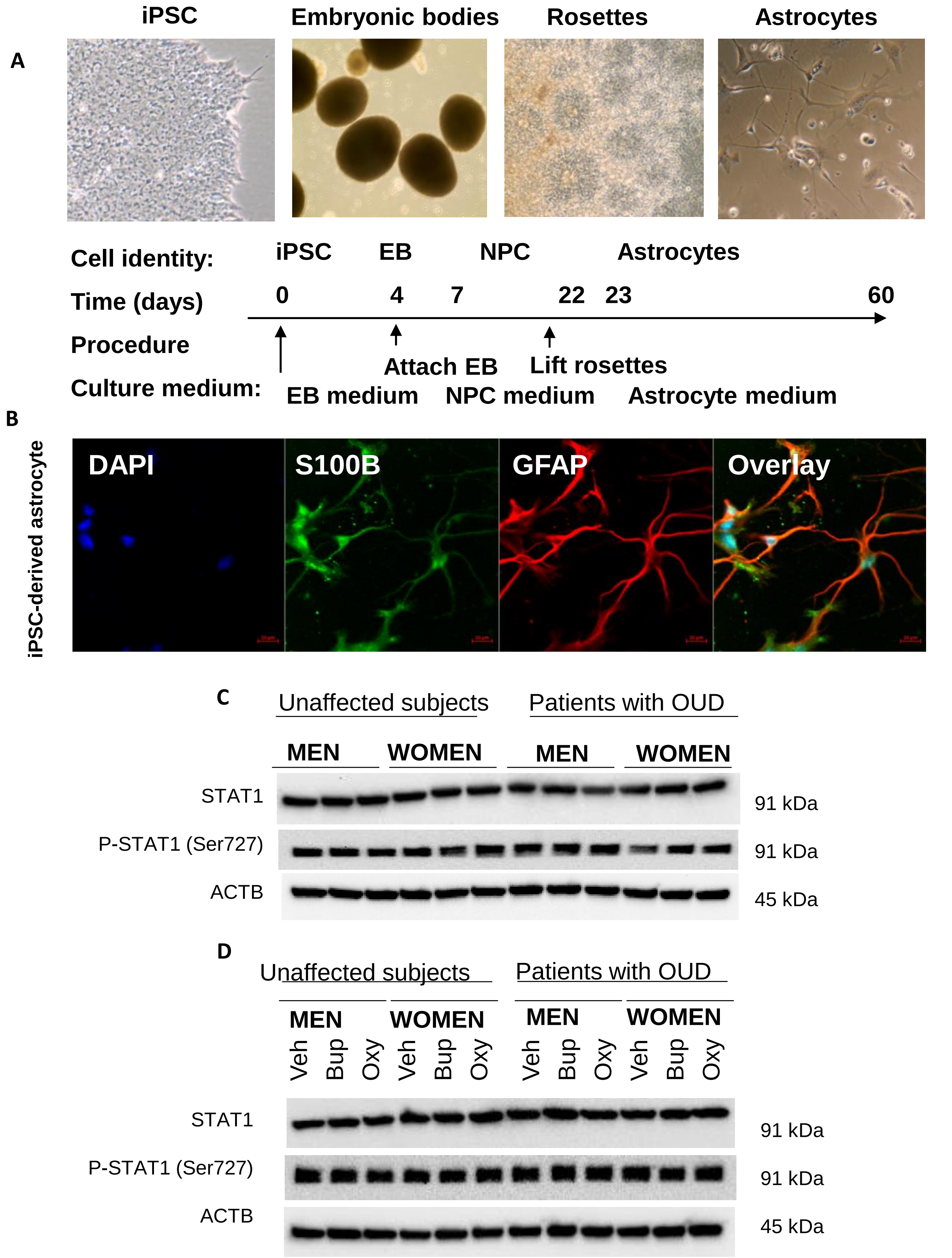
iPSC
Embryonic bodies
Rosettes
Astrocytes
A
Cell identity:
iPSC
EB
NPC
Astrocytes
Time (days)
0
4
7
22
23
60
Procedure
Attach EB
Lift rosettes
Culture medium:
EB medium
NPC medium
Astrocyte medium
B
iPSC-derived astrocyte
DAPI
S100B
GFAP
Overlay
C
Unaffected subjects
Patients with OUD
MEN
WOMEN
MEN
WOMEN
STAT1
P-STAT1 (Ser727)
ACTB
91 kDa
91 kDa
45 kDa
D
Unaffected subjects
Patients with OUD
MEN
WOMEN
MEN
WOMEN
Veh
Bup
Oxy
Veh
Bup
Oxy
Veh
Bup
Oxy
Veh
Bup
Oxy
STAT1
P-STAT1 (Ser727)
ACTB
91 kDa
91 kDa
45 kDa
