# Supplementary material for: Single cell transcriptomics reveals distinct transcriptional responses to oxycodone and buprenorphine by iPSC-derived brain organoids from patients with opioid use disorder
Source: Mol Psychiatry. 2022 Oct 27;29(6):1636–46. doi: 10.1038/s41380-022-01837-8 (PMC10588459; doi:10.1038/s41380-022-01837-8)
Supplement: Supplementary file 2 — Supplementary Figure and Table legend [file 41380_2022_1837_MOESM2_ESM.pdf]

Supplementary materials

Single cell transcriptomics reveals distinct transcriptional responses to oxycodone and buprenorphine by iPSC-derived brain organoids from patients with opioid use disorder

Ming-Fen Ho, Ph.D.[1,2*], Cheng Zhang, Ph.D.[2], Irene Moon, M.S[2], Xiujuan Zhu, Ph.D.[2], Brandon J. Coombes, Ph.D.[3], Joanna Biernacka, Ph.D.[3], Michelle Skime, M.S.[1], Tyler S. Oesterle, MD. M.P.H.[1], Victor M Karpyak, MD, Ph.D.[1], Kristen Schmidt, M.D.[4], Kate Gliske, Ph.D.[4], Quyen Ngo, Ph.D.[4], Cedric Skillon, M.D.[4], Marvin D. Seppala, M.D.[4] Hu Li, Ph.D.[2], and Richard M. Weinshilboum, M.D.[2*]

Fig. S1 A schematic outline of procedures used during the differentiation of iPSC-derived forebrain neurons. (B) Representative examples of staining for neuronal markers. Most iPSC-derived neurons (>90%) are glutamatergic excitatory neurons that express vesicular Glutamate Transporter 1 (VGLUT1). Approximately 5-10 % neurons are GABAergic neurons that express glutamate decarboxylase 1 (GAD1), and less than 1% neurons are dopaminergic neurons that express tyrosine hydroxylase (TH).

Fig. S2 (A) mRNA expression of three subtypes of opioid receptor (OPRM1, OPRK1, and OPRD1) in iPSC-derived forebrain organoids. (B) demographic and characteristics of the three male subjects included in the snRNA-seq study and cell lines for functional genomics studies. (C) bulk RNA-seq matrix of iPSC-derived forebrain organoids (n=3). (D) snRNA-seq matrix of iPSC-derived forebrain organoids. (E) UMAP plot demonstrates the distributions of cell in each treatment condition (V: vehicle, O: oxycodone, B: buprenorphine). (F) Percentage of cells from each subject and each treatment condition which were used to generate the UMAP plot as shown in Supplementary figure 2E.

Fig. S3 UMAP of cells colored by canonical marker genes list in supplementary table 2.

Fig. S4 (A) A schematic outline of procedures used during the differentiation of iPSC-derived forebrain astrocytes. (B) Representative examples of staining for astrocyte markers: S100B and GFAP. (C) Protein expression of STAT1 was determined using the iPSC-derived astrocyte from male and female OUD patients and unaffected controls (n=3 each group). The basal level of STAT1 protein expression showed no difference between OUD and unaffected controls. (D) Representative Western blot images demonstrate that STAT1 expression did not alter in response to oxycodone or buprenorphine treatment in iPSC-derived astrocytes from patients with OUD or unaffected controls. veh: vehicle, bup: buprenorphine, oxy: oxycodone.

Table S1. Primer sequences and antibodies used for experiments.

Table S2. Canonical marker genes to classify UMAP clusters 0–15.

Table S3. Pathway analysis was performed using the results derived from the differential gene expression analysis in each cell cluster. The values represent FDR. V: vehicle, B: buprenorphine, O: oxycodone.
